# Formula for the Preparation of the Ethereal Solution of Gun Cotton

**Published:** 1848-10

**Authors:** 


*Formula for the preparation of the Ethereal Solution of Gun Cotton.*—We have found considerable difficulty in preparing the gun cotton in a state to ensure its solubility in sulphuric ether. Our experiments would lead to the conviction, that the finest quality of gun cotton, which we have had no difficulty in preparing, is soluble, or nearly so, in that liquid. A gun cotton, of ready solubility and easy manufacture, may be prepared as follows: Take of nitric acid, sp. gr. 1.350 (the ordinary sp. gr. of commercial nitric acid) ℥ij.; sulphuric acid (commercial) ℥iv. Having mixed the acids in a glass vessel, stirring them with a glass rod, add immediately, of freshly carded cotton, ʒij. ℈ij., and digest for the period of fifteen minutes. The acid is now to be poured off the cottton, and the latter washed with water until litmus paper is not affected. The cotton is to be finally squeezed between the folds of a clean towel, to remove as much water as possible; teased out, and finally pressed between sheets of blotting paper, until quite dry, and instantly thrown into rectified sulphuric ether. The quantity of gun cotton thus formed is sufficient for about a pound of ether. It should form a transparent, colourless liquid, somewhat of the appearance of thin mucilage.

*Brit. and Am. Journ. of Med. Science.*

